# Evaluation of the Antiviral Activity of Sitagliptin-Glatiramer Acetate Nano-Conjugates against SARS-CoV-2 Virus

**DOI:** 10.3390/ph14030178

**Published:** 2021-02-24

**Authors:** Nabil A. Alhakamy, Osama A. A. Ahmed, Tarek S. Ibrahim, Hibah M. Aldawsari, Khalid Eljaaly, Usama A. Fahmy, Ahmed L. Alaofi, Filippo Caraci, Giuseppe Caruso

**Affiliations:** 1 Department of Pharmaceutics, Faculty of Pharmacy, King Abdulaziz University, Jeddah 21589, Saudi Arabia; nalhakamy@kau.edu.sa (N.A.A.); oaahmed@kau.edu.sa (O.A.A.A.); haldosari@kau.edu.sa (H.M.A.); uahmedkauedu.sa@kau.edu.sa (U.A.F.); 2 Center of Excellence for Drug Research and Pharmaceutical Industries, King Abdulaziz University, Jeddah 21589, Saudi Arabia; 3 Mohamed Saeed Tamer Chair for Pharmaceutical Industries, King Abdulaziz University, Jeddah 21589, Saudi Arabia; 4 King Fahd Medical Research Center, King Abdulaziz University, Jeddah 21589, Saudi Arabia; 5 Department of Organic chemistry, Faculty of Pharmacy, King Abdulaziz University, Jeddah 21589, Saudi Arabia; tmabrahem@kau.edu.sa; 6 Department of Pharmacy Practice, Faculty of Pharmacy, King Abdulaziz University, Jeddah 21589, Saudi Arabia; khalid-eljaaly@live.com; 7 Pharmacy Practice and Science Department, College of Pharmacy, University of Arizona, Tucson, AZ 85721, USA; 8 Department of Pharmaceutics, College of Pharmacy, King Saud University, Riyadh 11451, Saudi Arabia; ahmedofi@ksu.edu.sa; 9 Department of Drug and Health Sciences, University of Catania, 95125 Catania, Italy; 10 Oasi Research Institute—IRCCS, 94018 Troina, Italy

**Keywords:** glatiramer acetate, sitagliptin, COVID-19, SARS-CoV-2, 3CL protease, nanoparticles

## Abstract

The outbreak of the COVID-19 pandemic in China has become an urgent health and economic challenge. There is a current race for developing strategies to treat and/or prevent COVID-19 worldwide. Severe acute respiratory syndrome coronavirus 2 (SARS-CoV-2) is the strain of coronavirus that causes COVID-19. The aim of the present work was to evaluate the efficacy of the combined complex (nano-conjugates) of two FDA-approved drugs, sitagliptin (SIT) and glatiramer acetate (GA), against a human isolate of the SARS-CoV-2 virus. SIT-GA nano-conjugates were prepared according to a full three-factor bilevel (2^3^) factorial design. The SIT concentration (mM, X_1_), GA concentration (mM, X_2_), and pH (X_3_) were selected as the factors. The particle size (nm, Y_1_) and zeta potential (mV, Y_2_) were assessed as responses. Characterization of the optimized formula for the Fourier-transform infrared (FTIR) spectroscopy and transmission electron microscopy (TEM) was carried out. In addition, the half-maximal inhibitory concentration (IC_50_) in Vero-E6 epithelial cells previously infected with the virus was investigated. The results revealed that the optimized formula of the prepared complex was a 1:1 SIT:GA molar ratio at a pH of 10, which met the required criteria with a desirability value of 0.878 and had a particle size and zeta potential at values of 77.42 nm and 27.67 V, respectively. The SIT-GA nano-complex showed antiviral potential against an isolate of SARS-CoV-2 with IC50 values of 16.14, 14.09, and 8.52 µM for SIT, GA, and SIT-GA nano-conjugates, respectively. Molecular docking has shown that the formula’s components have a high binding affinity to the COVID 3CL protease, essential for coronavirus replication, paralleled by 3CL protease inhibition (IC_50_ = 2.87 µM). An optimized formulation of SIT-GA could guarantee both enhanced deliveries to target cells and improved cellular uptake. Further clinical studies are being carried out to validate the clinical efficacy of the optimized formulation against SARS-CoV-2.

## 1. Introduction

The recent outbreak of COVID-19 pneumonia in China has become an urgent health and economic challenge owing to its pandemic proportions [1]. COVID-19 pneumonia is caused by the new severe acute respiratory syndrome coronavirus 2 (SARS-CoV-2), belonging to the genus Betacoronavirus (β-CoV), one of the four coronavirus genera [2,3]. Although the coronavirus has been recognized since the 1930s, in the past two decades, two other deadly β-CoVs have burst onto the world scene, namely, SARS-CoV and MERS-CoV (which causes the Middle East respiratory syndrome); they have not had the extensive impact of SARS-CoV-2, however [4]. For viral‒host interactions, SARS-CoV and SARS-CoV-2 utilize ACE2 as a functional receptor, and MERS-CoV utilizes the DPP4 (dipeptidyl peptidase 4) receptor [5]. Developing antiviral agents such as viral inhibitors is one of the strategies for conquering the COVID-19 pandemic, as well as a strategy for vaccination. It is important to mention that the severity of the COVID-19 varies among infected patients and has been linked to the patients’ underlying state of health. For instance, diabetic patients who contract SARS-CoV-2 have had higher rates of severity and mortality [6]. Therefore, diabetes is an important risk factor for the severity of and mortality due to COVID-19. Cardiovascular diseases and hypertension, as well as diabetes, are the most prevalent cardiometabolic comorbidities in hospitalized COVID-19 patients, according to recent publications [7,8]. In Europe, the most frequent comorbidities of COVID-19 patients in intensive care units were hypertension, obesity, and diabetes. Recent studies suggest that obesity may be associated with increased COVID-19 severity even in younger patients [9,10].

Sitagliptin (SIT), a specific DPP4 inhibitor, may specifically reduce the excessive and prolonged cytokine responses observed in COVID-19 patients [11]. Interestingly, SIT treatment is associated with reduced mortality in hospitalized patients with type 2 diabetes and COVID-19 [12]. A potential immunomodulatory effect of SIT was suggested by a reduction in the plasma C-reactive protein and procalcitonin in patients treated with SIT [13]. SIT improves glycemic control by slowing the inactivation of incretin hormones, increasing insulin synthesis and release from pancreatic beta cells, and lowering glucagon secretion, all factors that can eventually improve the clinical outcomes of COVID-19 patients. In contrast, worse clinical outcomes were observed in COVID-19 patients with type 2 diabetes, and they might be attributed to the poor control of blood sugar levels. Age-dependent cellular and humoral immunity alterations could favor increased viral replication and a more prolonged inflammatory response, and these factors could be potentially responsible for the enhanced mortality outcome. Some of these changes may be reversed by the DPP4 inhibitor, SIT. DPP4 is a multifunctional glycoprotein that exists as an integral plasma membrane glycoprotein (i.e., anchored to varieties of cell surfaces) and as a soluble dimer in the plasma [14,15]. Interestingly, the up-regulation of soluble DPP4 plasma levels is considered a positive effect exerted by SIT [16]. Glatiramer acetate (GA), also known as Copaxone, is an immunomodulator drug used to treat multiple sclerosis [17]. It has also been recently associated with a lower risk of infections. One study in a multiple sclerosis population showed a salutary effect of interferon-β on human herpesvirus type 6 compared with control groups. The development of novel treatment strategies is required [18]. Recent studies suggest that GA can be protective against COVID-19 infection by rescuing natural killer cell activity [19].

The ability of nanostructures to interact with bacterial or viral microorganisms is rapidly revolutionizing different biomedical fields. The use of these nanostructures can efficiently enhance diagnostic and therapeutic approaches. For instance, nanoparticles can be utilized to improve drug delivery [20,21,22]. This is attributed to their unique physical properties, such as particle size, large surface area, large drug payloads, and other unique properties. The bioavailability and circulation time of a drug can be enhanced by optimized particle size, while a higher solubility can be achieved by increasing the surface-area-to-volume ratio. These benefits can be obtained by nanoparticulate drug delivery systems, which must be explored in order to achieve and/or improve therapeutic and diagnostic effects [23].

The focus of the present work was to investigate the synergistic antiviral activity of SIT-GA nano-conjugates in Vero-E6 epithelial cells previously infected with isolates of hCoV-19/Egypt/NRC-3/2020 virus (SARS-CoV-2). A full three-factor bilevel (2^3^) factorial design was first used for the preparation and optimization of SIT-GA nano-conjugates. The optimized SIT-GA nano-conjugates were then used to test their potential antiviral activity by performing experiments with infected Vero-E6 cells along with in vitro (cell-free) and in silico (molecular docking) experiments. A synergistic antiviral activity of SIT-GA nano-conjugates based, at least in part, on the ability of these nano-conjugates to inhibit 3CL protease activity, is presented.

## 2. Results

### 2.1. Experimental Design of SIT-GA Nano-Conjugates

#### 2.1.1. Analysis of the Factorial Design

Analysis of variance (ANOVA) was utilized to assess the main effects of the studied variables on each response (runs shown in Table 1).

For both responses, the predicted R^2^ values were in rational agreement with the adjusted R^2^ values. Adequate precision was greater than 4 (Table 2), confirming that the model could be successfully employed to explore the experimental design space [24,25].

#### 2.1.2. Effect of Variables on Particle Size (Y_1_)

The particle size of the prepared SIT-GA nano-conjugates ranged from 78.7 ± 1.0 to 276.5 ± 3.1 nm (Table 1).

Based on the analysis of the factorial design, the factorial model with the main effects process order was significant (model F-value = 88.25; *p* = 0.0004). There is only a 0.04% chance that an F-value could occur due to noise. The equation describing the main effects in terms of coded factors was generated as follows (Equation (1)):

Y_1_ = 174.00 + 28.08 X_1_ + 63.92 X_2_ − 3.76 X_3_ (1)

**Figure 1:**
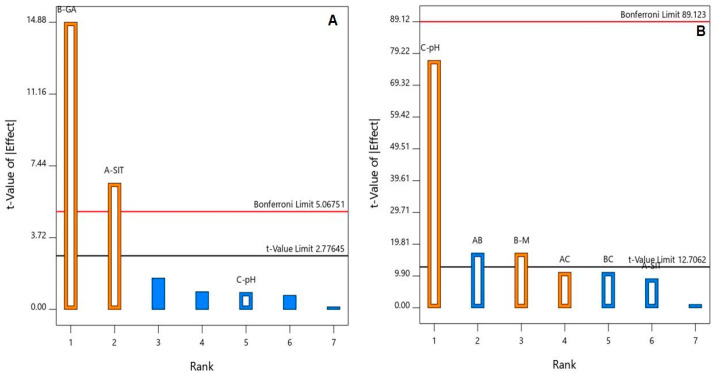
Standardized Pareto chart for the (**A**) particle size and (**B**) zeta potential of the SIT-GA nano-conjugates.

The ANOVA, using the Type III partial sum of squares, showed that both SIT (X_1_) and GA (X_2_) molar concentrations had a significant positive effect on the particle size (*p* = 0.0028 and 0.0001, respectively). This positive effect is evidenced by the positive sign of the coefficients of both X_1_ and X_2_ and graphically illustrated in the Pareto chart in Figure 1A.

**Figure 2:**
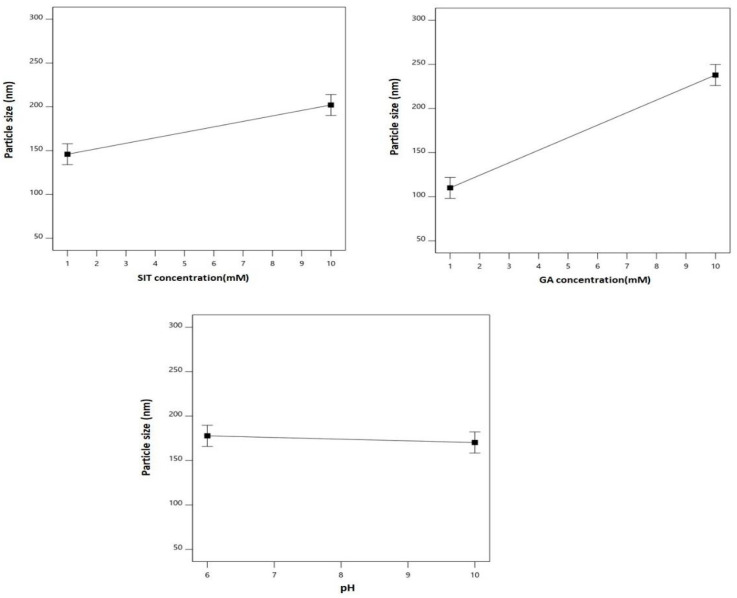
Main effects of the SIT concentration (X_1_), GA concentration (X_2_), and pH (X_3_) on the particle size of the SIT-GA nano-conjugates.

Figure 2 graphically illustrates the individual effects of the assessed variables on the particle size. As clearly depicted, the size increased with increases in both SIT and GA concentrations.

#### 2.1.3. Effect of Variables on the Zeta Potential (Y_2_)

All the prepared SIT-GA nano-conjugates exhibited a positive zeta potential ranging from 6.2 ± 0.1 to 33.3 ± 0.7 (Table 1). Based on the analysis of the factorial design, the factorial model with the two-factor interaction (2FI) process order showed significance at the set level (model F-value = 1138.33; *p* = 0.0227). There is a liability of only 2.27% that an F-value could be due to noise. The equation expressing the main effects and interactions using coded factors was generated as follows (Equation (2)):

Y_2_ = 20.41 − 1.17 X_1_ + 2.21 X_2_ + 10.01 X_3_ − 2.21 X1X2 + 1.43 X_1_X_3_ − 1.43 X_2_X_3_ (2)

An ANOVA, using the Type III partial sum of squares, revealed a significant impact of both GA concentrations (X_2_, *p* = 0.0374) and pH (X_3_, *p* = 0.0083) on the zeta potential, as shown in the Pareto chart in Figure 1B. In addition, the interaction term X_1_X_2_ (*p* = 0.0374) corresponding to the interaction between the SIT and GA concentrations was significant at the same level.

**Figure 3:**
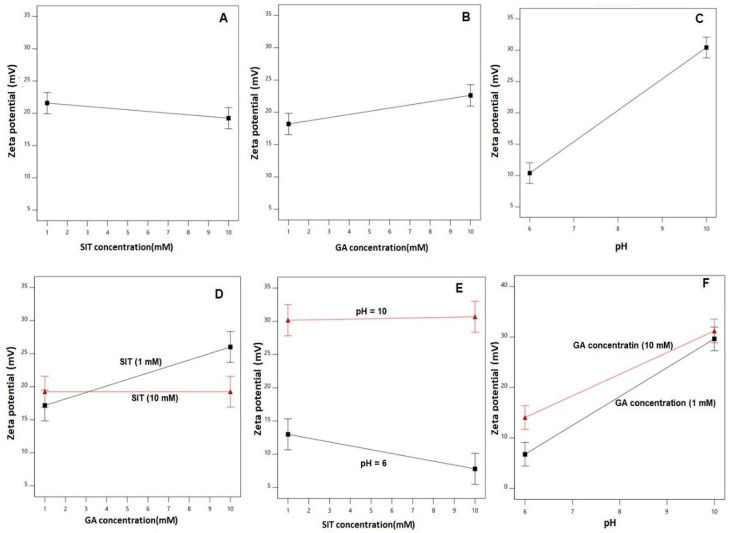
Main effects (**A** to **C**) and interactions (**D** to **F**) of the SIT concentration (X_1_), GA concentration (X_2_), and pH (X_3_) on the zeta potential of the SIT-GA nano-conjugates.

The main effects of the studied factors and the 2FI between these factors on the zeta potential are graphically represented in Figure 3.

As clearly shown, the zeta potential values increased with increasing GA concentration and pH. The effect of the pH was more prominent on the zeta potential, as demonstrated by its higher coefficient in the coded equation. The effect of the GA was prominent at a lower drug concentration rather than at the higher one, as depicted in the interaction graph (Figure 3D).

### 2.2. Selection of the Optimized SIT-GA Nano-Conjugates

The optimal SIT-GA nano-conjugates were selected based on the set goals for the responses, and the desirability function was computed. It was found that the nano-conjugates formulated using a SIT concentration of 1.0 mM and a GA concentration of 1.0 mM at a pH of 10 met the required criteria with a desirability value of 0.878. Therefore, the optimized formula was picked for performing the biological analyses. The predicted formulation was prepared and assessed for particle size and zeta potential, with results of 77.42 nm and 27.67 mV, respectively (Table 3).

The results were in good agreement with the predicted values (78.24 nm and 27.17 mV, respectively), with a residual error percentage of 1.06% and 1.84%, respectively (Table 3). The optimal SIT-GA nano-conjugates showed a polydispersity index value of 0.312 that indicates the average uniformity of particle distribution.

### 2.3. Fourier-Transform Infrared Spectroscopy Investigation of the Optimized SIT-GA Nano-Complex

**Figure 4:**
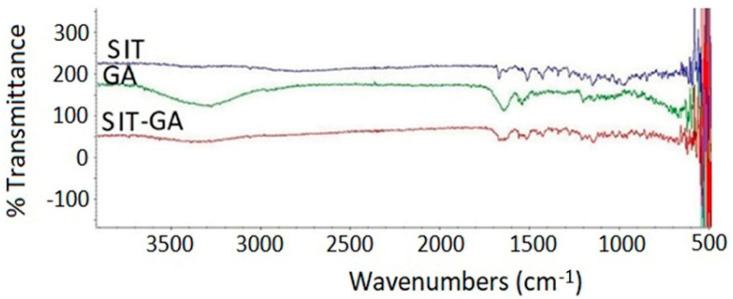
Fourier-transform infrared (FTIR) spectra of SIT, GA, and a SIT-GA nano-complex.

The SIT base form showed characteristic band regions that can be assigned as follows: 3049 cm^−1^ for the aromatic C-H stretching, 1650 to 1690 cm^−1^ for the amidic C=O bond stretching, 1630 cm^−1^ for the imine C=N bond, 1580 cm^−1^ for the N-H bending vibration (N–H), 1465 cm^−1^ for the C-H bending of the methylene group, and the vibrations at 1000 to 1400 cm^−1^ are related to fluoride (C–F). The main characteristic of the SIT infrared spectra is the absence of broadband of OH stretching at 3000 to 3500 cm^−1^ due to water molecules of SIT phosphate (Figure 4).

There is a very clear broadband peak of GA spectra at 3200 to 3500 cm^−1^ due to multiple –NH_2_ and –COOH groups of amino acids. GA also showed a broad peak at 1630 to 1710 cm^−1^ owing to the carbonyl of the COOH group. SIT-GA FTIR showed that there is a complete absence of a broad peak at 3200 to 3500 cm^−1^. Additionally, a sharp decrease in the intensity of the characteristic function group peaks for both GA and SIT at 1500 to 1700 cm^−1^ indicates that an interaction between SIT and GA results in an enhanced stabilization of our formula [26].

### 2.4. Transmission Electron Microscope (TEM) Investigation of the Optimized SIT-GA Nano-Conjugates

**Figure 5:**
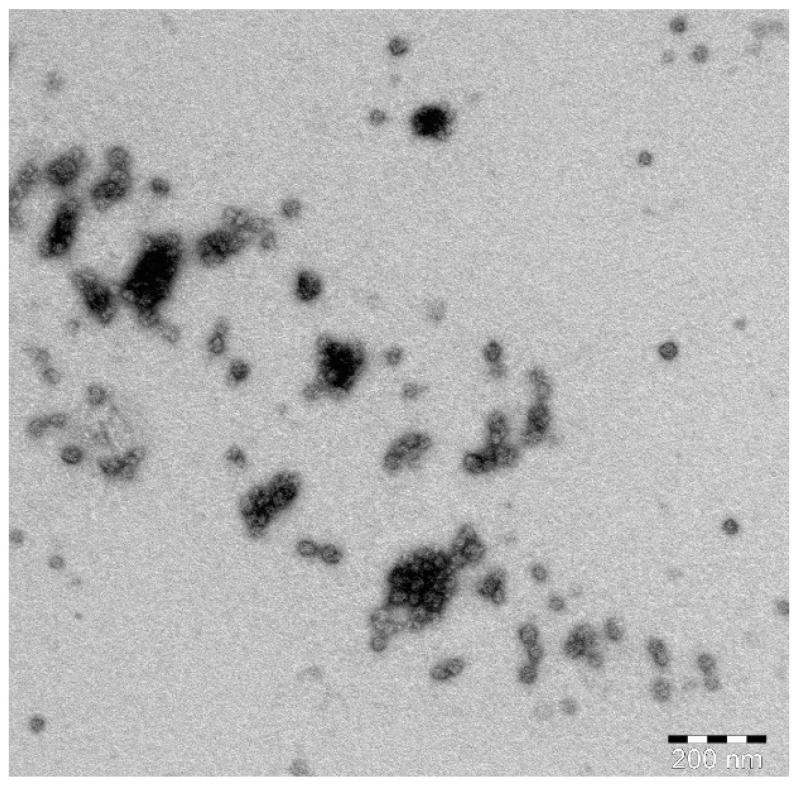
TEM image of the optimized SIT-GA nano-conjugates.

TEM images of the optimized SIT-GA nano-conjugates showed an almost spherical structure with some aggregates that could be resulted from the drying process during the preparation of the sample (Figure 5).

### 2.5. In Vitro Antiviral Screening Activity 

**Figure 6:**
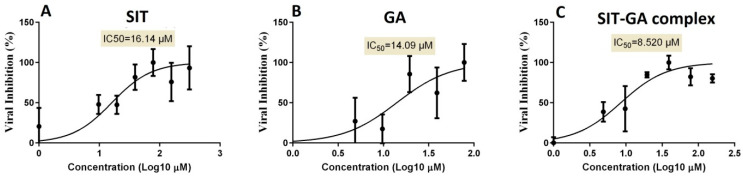
Determination of the half-maximal inhibitory concentration (IC_50_) of (**A**) SIT, (**B**) GA, and (**C**) SIT-GA nano-conjugates against SARS-CoV-2 (Vero-E6 infected cells).

To identify the proper concentrations for defining the antiviral activity of the SIT, GA, and SIT-GA nano-conjugates, the half-maximal cytotoxic concentration (CC_50_) was calculated by the crystal violet assay for each individual experimental condition. The half-maximal inhibitory concentration (IC_50_) values, based on the measurements obtained by employing the crystal violet assay, were calculated using the non-linear regression analysis of GraphPad Prism software (version 5.01, San Diego, CA, USA) by plotting the log inhibitor versus the normalized response (variable slope). The antiviral screening revealed that the tested drugs exhibited promising in vitro antiviral activity against SARS-CoV-2 (Vero-E6 infected cells). It was observed that the SIT-GA combination exhibited an enhanced (synergistic) effect (IC_50_ = 8.52 µM) compared to SIT (IC_50_ = 16.14 µM) or GA (IC_50_ = 14.09 µM) (Figure 6). 

### 2.6. In Vitro Mpro, 3CL Protease Inhibition Test

**Figure 7:**
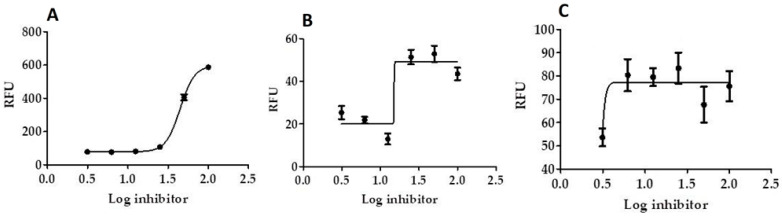
Inhibition of 3CL protease enzyme activity by SIT (**A**), GA (**B**), and (**C**) SIT-GA nano-conjugates. RFU = relative fluorescence units.

Results show that in vitro Mpro, 3CL protease inhibition of SIT-GA nano-conjugates was significantly enhanced (IC_50_ = 2.876 ± 0.21 µM, *p* < 0.001), compared with the individual components SIT (IC_50_ = 109.926 ± 0.94 µM; Figure 7A) and GA (IC_50_ = 26.732 ± 0.65 µM; Figure 6B) against GC376 (standard 3CL protease enzyme inhibitor; IC_50_ = 0.400 ± 0.23 µM).

### 2.7. Molecular Docking and Virtual Screening Study

**Figure 8:**
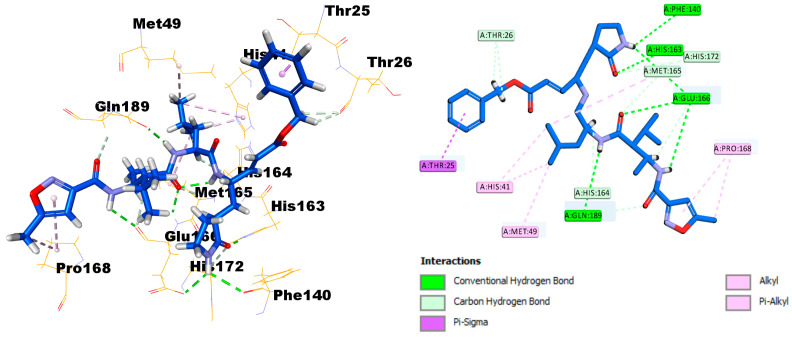
Docking and binding mode of ligand N3 into the active site of the SARS-CoV-2 Mpro structure (PDB ID: 6LU7).

The X-ray crystal structure coordinates of the SARS-CoV-2 main protease (Mpro) were retrieved from the protein data bank (PDB ID: 6LU7). To investigate the binding affinity between the protein and SIT, the Discovery Studio software package was used. First, validation of the docking protocol was undertaken by redocking of the ligand N3 in the Mpro crystal structures. The root‒mean‒square deviation value was less than 0.853 (<2), reflecting that one could place great trust in the produced docking results. It is clearly established that the N3 ligand, as shown in Figure 8, engaged with six hydrogen bonds with the amino acid residues Phe140, His163, Clu166 (three H.B.), and Gln189, in addition to many hydrophobic interactions.

SIT can strongly bind to the substrate-binding pocket of the SARS polymerase structure (PDB ID: 6LU7) and showed significant inhibition of the C.D.O.C.K.E.R. energy of SIT (−29.9792) and the C.D.O.C.K.E.R. interaction energy (−53.5594), as compared with the standard Ligand N3 (the C.D.O.C.K.E.R. energy was −70.8463, while the C.D.O.C.K.E.R. interaction energy was −79.0435). SIT engaged with four hydrogen bonds with the amino acid residues Phe140, Ser144, His163, and Glu166, in addition to three halogen interactions with Leu140, His164, and Thr190, and many hydrophobic interactions with Cys145, Met165, Glu166, and Gln189 (Figure 8).

## 3. Discussion

When designing formulations, it is important to properly consider the cellular uptake [27,28,29]. This is because of several physical properties of the formulations that are linked directly to the uptake property, such as the therapeutic load, that eventually affect the optimal dose. However, the efficacy of the uptake can be controlled by cellular membrane characteristics, along with other physical properties of the nanoparticles [29]. Pointing out the formulation and process parameters that could influence the drug delivery system characteristics is of utmost importance in pharmaceutical formulations. According to this scenario, a factorial design is helpful because it can analyze the effect of various factors jointly. Factorial designs can address more than one inquiry in the same study with an adequate number of experimental runs. Multiple factors are manipulated or allowed to change to allow for examination of their main effects simultaneously. Additionally, such a design could provide insight regarding the possible interactions that could be detected only upon examining the independent variables in combination. In this study, the factors and their corresponding levels were chosen based on the results of preliminary trials. The ANOVA was utilized to assess the main effects of the studied variables on each response. For both responses, the predicted R^2^ values were in rational agreement with the adjusted R^2^ values (Table 2). 

Drug repurposing (also known as drug repositioning) represents an unconventional drug discovery approach to investigate new therapeutic benefits of existing/available drugs. In the last decade, this approach has been considered to fight infections and other diseases, including COVID-19 [30,31]. In this regard, the present work explored the potential antiviral activity of SIT and GA, two drugs normally used for the treatment of type 2 diabetes mellitus [32] and multiple sclerosis [17], respectively. Recent studies suggest that each of these drugs possess a good therapeutic potential against COVID-19 [12,19], although no studies have been conducted to evaluate the antiviral activity of SIT-GA nano-conjugates. It is well-known that the particle size could exert a significant effect on the biological performance of the nanosized particulate delivery systems. As clearly shown, the particle size increases with increases in both SIT and GA concentrations (Figure 2). This could be related to an increased chance for ionic interaction and aggregation of the SIT-GA nano-complex. In addition, the increase in particle size is related to the increased frictional forces of the entrapped SIT and GA that accrue as their concentrations increase. This reduces their chance of escape and leads to increased particle size. Therefore, nanoparticle size is considered a major determinant of cellular uptake, with approximately 50 nm in diameter being optimum for non-phagocytic cells. Various ligands (proteins or peptides) can be used to enhance cellular uptake, such as the HIV-derived TAT peptide, which facilitates cellular penetration [33,34]. It is also critical to recall that nanoparticles’ surface charge comes with an influence that can show whether nanoparticles are able or not to cross the cell membrane with its negative charge. The reason why the overall nanoparticles’ surface charge is increased was to find a result of an increase in the uptake by the cellular membranes [35,36]. The cellular internalization could take place through different mechanisms, including clathrin-mediated endocytosis, macropinocytosis, caveolar-mediated endocytosis, and phagocytosis. The mechanism by which nanoparticles are internalized is also related to nanoparticles’ size [37]. An indication is given by the zeta potential for the charge stabilization for the systems of nano-particulates [38,39]. The net positive charge of the SIT-GA nano-complex facilitates its interaction with the negatively charged phospholipids of the cell membrane, improving cellular internalization. It has also recently been demonstrated that the shape of the nanoparticles is a determining factor of the mechanism of uptake. Therefore, the knowledge of these aspects is of utmost importance in the engineering of nanoparticles targeted to specific microenvironments.

The current study signified that novel outcomes should be used as guiding factors or recommendations during the repurposing of the two FDA-approved drugs and their nano-formulations that were expected to be effective against COVID-19. Our results revealed that SIT, GA, and SIT-GA nano-conjugates had potent antiviral activities against SARS-CoV-2 in Vero-E6 cells, with IC50 values of 16.14, 14.09, and 8.52 µM, respectively (Figure 6). We showed that SIT and GA have antiviral activity against an isolate of SARS-CoV-2 that is synergized two-fold when a combination of the two drugs is applied in an optimized nano-complex formulation (SIT-GA nano-conjugates). Additionally, SIT-GA nano-conjugates showed a significantly enhanced ability to inhibit 3CL protease compared to individual drugs (Figure 7). This enhanced activity could be of great relevance since 3CL protease is able to hydrolyze viral polyproteins to produce functional proteins and is essential for coronavirus replication [40]. Of note, the results of the in silico studies demonstrated a strong binding affinity of SIT to the viral main protease receptor of SARS-CoV-2 (Figure 8), which could represent a mechanism linked to its observed antiviral activity.

Overall, our data suggest a high therapeutic potential of SIT-GA nano-conjugates as a novel pharmacological tool against COVID-19. It would be interesting to evaluate in future studies the immunomodulatory action of SIT-GA nano-conjugates and the impact of this novel pharmacological tool on pro-inflammatory cytokine release in experimental models of COVID-19.

## 4. Materials and Methods

### 4.1. Materials

Glatiramer acetate (Copaxone; Teva Pharmaceuticals, Parsippany, New Jersey, USA) in samples of 20 mg/mL (dosage form: injection, solution) was donated by Sharon G. Lynch, MD, Department of Neurology, University of Kansas Medical Center (Kansas City, KS, USA). Sitagliptin was a gift from the Jamjoom Pharmaceuticals Company (Jeddah, Saudi Arabia). All the remaining materials, unless specified otherwise, were supplied by Thermo Fisher Scientific Inc. (Pittsburgh, PA, USA) or Sigma-Aldrich Corporate (St. Louis, MO, USA). All the materials were used as supplied.

### 4.2. Experimental Design for the Preparation and Optimization of SIT-GA Nano-Conjugates

SIT-GA nano-conjugates were prepared according to a full three-factor bilevel (2^3^) factorial design using Design-Expert^®^ software version 12 (Stat-Ease, Inc., Minneapolis, Minnesota, U.S.A.). Two formulation factors and one processing factor were selected as independent variables, namely, SIT concentration (mM, X_1_), GA concentration (mM, X_2_), and pH (X_3_). The particle size (nm, Y_1_) and zeta potential (mV, Y_2_) were assessed as responses (dependent variables), as shown in Table 4.

Ionic interaction occurs between the negatively and positively charged essential function groups of SIT and GA, respectively, according to the molar ratio.

A total of eight formulations were yielded by combining the different levels of the independent variables (Table 1). The significance of the main effects of the variables on the studied responses was determined by using the ANOVA test at a *p*-value of less than 0.05. The equations representing the selected factorial model for each response were generated in terms of coded factors, and the main effect plots were also generated. The desirability function that merges all the measured responses to predict the optimum levels of the independent variables was computed to select the optimal formulation. Minimizing the particle size and maximizing the magnitude of the zeta potential were the goals set for optimizing the proposed formulations (Table 4).

#### 4.2.1. Preparation of the SIT-GA Formulations

The SIT-GA formulations were designed and prepared according to the experimental design (Table 1). Different concentrations of SIT and GA were placed in 20 mL of 0.01 M phosphate-buffered saline (PBS) with different pH levels and then vortexed for 2 min [26].

#### 4.2.2. Determination of the Particle Size and Zeta Potential

The prepared nanoparticles of the SIT-GA formulations were dispersed in water. The particle size and zeta potential of the nanoparticles were then measured by a particle size analyzer (Zetatrac; Microtrac, Inc., Montgomeryville, PA, USA). To determine the zeta potential and particle size, 1 mL of the prepared complexes was diluted into 10 mL of the same buffer. The average particle size and zeta potential were determined from three replicate readings.

#### 4.2.3. Optimization of the SIT-GA Preparations

The two-way ANOVA and multiple-response optimization were applied in the statistical analysis of the results. A comparison of zeta potential and particle size between the predicted optimum formulation and the actual prepared formulation was carried out to validate the results.

#### 4.2.4. FTIR Spectroscopy Investigation of the Optimized SIT-GA Complex

FTIR analysis was utilized to investigate the interaction between SIT and GA spectra. They were measured at between 4000 and 400 cm^-1^ using an FTIR spectrophotometer (Nicolet iZ10; Thermo Fisher Scientific, Waltham, MA, USA).

#### 4.2.5. TEM Investigation of the Optimized SIT-GA Nano-Conjugates

One drop of the optimized SIT-GA nano-conjugates dispersion was spread on a carbon grid, stained with phosphotungistic acid, dried, and then investigated utilizing TEM (JEM-1011: JEOL, Tokyo, Japan).

### 4.3. Determination of the CC_50_ and IC_50_ Values

Vero-E6 cells were maintained in Dulbecco’s modified Eagle’s medium (DMEM) containing 10% fetal bovine serum (FBS) (Invitrogen, Carlsbad, CA, U.S.A.) and a 1% penicillin/streptomycin (pen/strep) antibiotic mixture at 37 °C in 5% CO_2_. To generate virus stock, cells were distributed into tissue culture flasks 24 h prior to infection with the hCoV-19/Egypt/NRC-3/2020 isolate [41] (GISAID Accession Number: EPI_ISL_430820) at a multiplicity of infection (MOI) of 0.1 in the infection medium (DMEM containing 2% FBS, 1% pen/strep, and 1% l-1-tosylamide-2-phenyl ethyl chloromethyl ketone (TPCK)-treated trypsin). Two hours later, the infection medium containing the virus inoculum was removed and replaced with a fresh infection medium, and this was incubated for three days. At the indicated time point, the cell supernatants were collected and centrifuged for 5 min at 2500 rpm to remove small particulate cell debris. 

**Figure 9:**
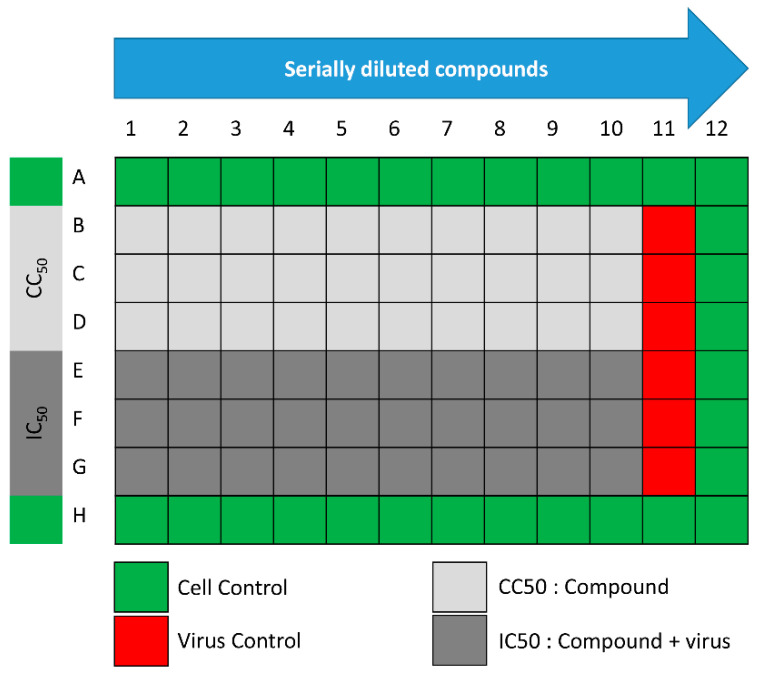
Schematic representation of the 96-well plates used for IC_50_ and half-maximal cytotoxic concentration (CC_50_) determinations.

The assay was performed according to the procedure that was previously described with minor modifications [31]. In 96-well tissue culture plates, 2.4 × 10^4^ Vero-E6 cells were distributed in each well and incubated overnight in a humidified (37 °C) incubator under 5% CO_2_ conditions. The cell monolayers were then washed once with 1× PBS and subjected to virus adsorption for 1 h at room temperature (RT). The cell monolayers were further overlaid with 50 μL of DMEM containing different concentrations of SIT, GA, or SIT-GA nano-conjugates. Following an incubation at 37 °C and 5% CO_2_ for 72 h, the cells were fixed by using 100 μL of 4% paraformaldehyde for 20 min and stained with 0.1% crystal violet in distilled water for 15 min at RT. The crystal violet dye was then dissolved using 100 μL of absolute methanol per well, and the optical density of the color was measured at 570 nm using an Anthos Zenyth 200rt plate reader (Anthos Labtec Instruments, Heerhugowaard, Netherlands). The IC50 of the compound is that required to reduce the virus-induced cytopathic effect by 50%, relative to the virus control. For the assessment of the CC50, the stock solutions (dimethyl sulfoxide 10% in ddH_2_O) of the test compounds were diluted (obtaining the working solutions) by using DMEM. At this point, the extract cytotoxic activity of each compound was measured through the same method described above (Figure 9).

### 4.4. In Vitro 3CL Protease Inhibition Test

A fluorescent substrate harboring the cleavage site (indicated by the arrow ↓) of SARS-CoV-2 Mpro (Dabcyl-KTSAVLQ↓SGFRKME-Edans) (BPS Bioscience, San Diego, CA, USA), 3C-like protease (SARS-CoV-2 3CL protease) (GenBank Accession No. YP_009725301, a.a. 1-306 (full length), expressed in an *Escherichia coli* expression system, MW 77.5 kDa), and a buffer composed of 20 mM tris(Hydroxymethyl)aminomethane (TRIS), 100 mM NaCl, 1 mM ethylenediaminetetraacetic acid (EDTA), 1 mM dithiothreitol (DTT) at a pH of 7.3 were used for the inhibition assay. A GC376 3CL protease inhibitor, MW 507.5 Da, was used as a control. In the fluorescence resonance energy transfer (FRET)-based cleavage assay, the fluorescence signal of the Edans generated due to the cleavage of the substrate by the 3CL protease was monitored at an emission wavelength of 460 nm with excitation at 360 nm, using an Flx800 fluorescence spectrophotometer (BioTek, Winooski, VT, USA) [42]. Initially, 30 µL of diluted SARS-CoV-2 3CL protease at the final concentration of 15 ng was pipetted into a 96-well plate containing a pre-pipetted 10 µL of the test compounds at concentrations ranging from 100 µg/mL to 1.562 µg/mL. The mixture was incubated for 30 min at RT with slow shaking. Afterward, the reaction was initiated by the addition of 10 µL of the substrate dissolved in the reaction buffer to a final volume of 50 μL, at a concentration of 40 μM, incubated for 4 h at RT with slow shaking. The plates were sealed. The fluorescence intensity was measured in a Spark^®^ multimode microplate reader (Tecan Group Ltd., Seestrasse, Maennedorf, Switzerland) capable of excitation at a wavelength of 360 nm and detection of emissions at a wavelength of 460 nm.

### 4.5. Docking Studies 

#### 4.5.1. Optimization of Target Compounds 

The presented molecular docking investigation was performed through the Molecular Operating Environment (MOE) platform. Both investigated ligands were build using the M.O.E. builder module and then energy-minimized via the MMFF-mediated partial charges and force field throughout the conjugate-gradient method of 2000 steps with a gradient of 1 × 10^−3^ Kcal/Å [43]. Prepared and minimized ligands were then saved as Molecular Database chemical file format to be utilized within the molecular docking protocol [44].

#### 4.5.2. Docking of the Target Molecules to the Active Binding Site of the Crystallographic Structure of Mpro (PDB ID: 6LU7)

Discovery Studio 2.5 software (Accelrys Software, Inc., San Diego, CA, USA) was used for the docking analysis. The fully automated docking tool used the “Dock ligands (C.D.O.C.K.E.R.)” protocol running on an Intel^®^ Core^TM^ i32370, CPU 2.4 GHz, 2GB Memory RAM, Windows 7.0. The X-ray crystallographic structure of Mpro complexed with the N3 ligand was obtained from the Protein Data Bank (PDB ID: 6LU7). The enzyme was prepared for docking studies via the automatic protein preparation module that was used for applying the C.H.A.R.M.M. force field. The binding site sphere had been defined automatically by the software [44]. Next, the above-prepared receptor was given as the input for the “input receptor molecule” parameter in the C.D.O.C.K.E.R. protocol parameter explorer. The obtained poses were studied, and the poses showing the best ligand–H.D.A.C. interactions were chosen and employed for the calculations of the C.D.O.C.K.E.R. energy (protein-ligand interaction energies). Finally, the receptor–ligand interactions of the complexes were investigated in 2D and 3D styles.

### 4.6. Statistical Analysis

The statistical analysis was performed using the IBM SPSS^®^ statistical software (Ver. 25, S.P.S.S. Inc., Chicago, IL, USA). Tukey’s post hoc test, along with one- or two-way ANOVA, was applied in case of multiple comparisons. Each set of experiments was performed at least four times before evaluating the results. *p*-values lower than 0.5 were considered to be significant.

## 5. Conclusions

In the present study, a full three-factor bilevel (2^3^) factorial design was used for the preparation of SIT-GA nano-conjugates as well as for their optimization, aiming at minimizing the size of nanoparticles and maximizing the zeta potential. The in vitro experiments carried out by using Vero cells infected with the virus showed the synergistic antiviral potential of SIT-GA nano-conjugates against a human isolate of SARS-CoV-2. This antiviral activity could depend on the ability of SIT-GA nano-conjugates to inhibit 3CL protease, as suggested by the results related to the in vitro (cell-free) and in silico (molecular docking) results. The use of an optimized formulation of SIT-GA could guarantee both enhanced deliveries to target cells and improved cellular uptake. Further clinical studies are being carried out to estimate the efficiency of the optimized formulation against SARS-CoV-2.

## Figures and Tables

**Table 1:** Experimental runs and the observed responses of the SIT-GA nano-conjugates prepared according to a 2^3^ factorial design.

Experimental Run Number	Independent Variables			Particle Size ± S.D.	Zeta Potential ± S.D.
	SIT Concentration (mM)	GA Concentration (mM)	pH		
**F-1**	1	10	6	220.8 ± 3.6	18.7 ± 0.2
**F-2**	10	1	10	147.8 ± 1.2	32.2 ± 0.9
**F-3**	1	1	6	77.4 ± 0.9	7.3 ± 0.1
**F-4**	10	10	10	247.7 ± 2.9	29.1 ± 0.6
**F-5**	10	10	6	276.5 ± 3.1	9.4 ± 0.2
**F-6**	1	10	10	206.7 ± 2.6	33.3 ± 0.7
**F-7**	10	1	6	136.3 ± 1.6	6.2 ± 0.1
**F-8**	1	1	10	78.7 ± 1.0	27.1 ± 1.1

Abbreviations: SIT = sitagliptin; GA = glatiramer acetate.

**Table 2:** Statistical analysis output of response data of 2^3^ factorial design used for the formulation of SIT-GA nano-conjugates.

Responses	Process Order	*p*-Value	R^2^	Adjusted R^2^	Predicted R^2^	Adequate Precision	Significant Factors and Interactions
Y_1_: particle size (nm)	Main effects	0.0004	0.9851	0.9740	0.9405	22.28	X_1_, X_2_
Y_2_: zeta potential (mV)	2FI	0.0227	0.9999	0.9990	0.9906	77.86	X_2_, X_3_, X_1_X_2_

Abbreviations: SIT = sitagliptin; GA = glatiramer acetate; 2FI = two-factor interaction.

**Table 3:** Optimized variables levels of the optimal SIT-GA nano-conjugates with predicted and observed values of the responses.

Variables	X1: SIT Concentration (mM)	X2: GA Concentration (mM)	X3: Hydrating Buffer pH
**Optimum values**	1.0	1.0	10
	**Predicted value**	**Observed value**	**Error %**
**Particle size (nm)**	78.24	77.42	1.06
**Zeta potential (mV)**	27.17	27.67	1.84

Abbreviations: SIT = sitagliptin; GA = glatiramer acetate.

**Table 4:** Independent variables and responses used in a 2^3^ full factorial experimental design for the formulation and optimization of SIT-GA nano-conjugates.

Independent Variables	Levels	
	(‒1)	(+1)
X_1_: SIT concentration (mM)	1	10
X_2_: GA concentration (mM)	1	10
X_3_: pH	6	10
**Responses**	**Desirability constraints**	
Y_1_: particle size (nm)	Minimize	
Y_2_: zeta potential (mV)	Maximize	

Abbreviations: SIT = sitagliptin; G.A., glatiramer acetate.

## Data Availability

Data are available within the article or from the corresponding author upon reasonable request.
